# GutBugDB: a web resource to predict the human gut microbiome-mediated biotransformation of biotic and xenobiotic molecules

**DOI:** 10.1017/gmb.2024.15

**Published:** 2025-01-09

**Authors:** Usha Longwani, Ashok K. Sharma, Aditya S. Malwe, Shubham K. Jaiswal, Vineet K. Sharma

**Affiliations:** MetaBioSys Lab, Department of Biological Sciences, Indian Institute of Science Education and Research, Bhopal, Madhya Pradesh, India

**Keywords:** biotransformation, database, human gut microbiome, machine learning, xenobiotic metabolism

## Abstract

There has been a growing recognition of the significant role played by the human gut microbiota in altering the bioavailability as well as the pharmacokinetic and pharmacodynamic aspects of orally ingested xenobiotic and biotic molecules. The determination of species-specific contributions to the metabolism of biotic and xenobiotic molecules has the potential to aid in the development of new therapeutic and nutraceutical molecules that can modulate human gut microbiota. Here we present “GutBugDB,” an open-access digital repository that provides information on potential gut microbiome-mediated biotransformation of biotic and xenobiotic molecules using the predictions from the GutBug tool. This database is constructed using metabolic proteins from 690 gut bacterial genomes and 363,872 protein enzymes assigned with their EC numbers (with representative Expasy ID and domains present). It provides information on gut microbiome enzyme-mediated metabolic biotransformation for 1439 FDA-approved drugs and nutraceuticals. GutBugDB is publicly available at https://metabiosys.iiserb.ac.in/gutbugdb/.

## Introduction

An extensive and diverse microbial population, which is vital for human health, is found in the gastrointestinal tract of human (Sharma et al., 2017). Comprising more than 10,000 billion microbial cells from roughly 4600 different bacterial species, the human gut microbiota (HGM) constitutes the largest and most diverse community among all other microbial communities colonising the human body (Guinane and Cotter, 2013; Almeida et al., 2021). HGM thus provides vast metabolic capabilities to the host to metabolise orally ingested drugs/xenobiotics as well as dietary bioactive components (Gentile and Weir, 2018). Many bioactive dietary components such as polyphenols, pigments, and oligosaccharides have antioxidants, antiestrogenic, anti-inflammatory, immunomodulatory, and anti-carcinogenic properties and are also formulated as nutraceuticals that can modulate gut microbiota by favouring the growth of beneficial commensal gut microbes (Cencic and Chingwaru, 2010). Thus, understanding and predicting the metabolism of biotic and xenobiotic molecules by gut microbiota is much needed (Jaiswal et al., 2021).

The ability of HGM to metabolise biotic and xenobiotic substrates can be attributed to the metabolic potential of enzymes that can promiscuously catalyse substrates with structural similarities to their native substrates (Khersonsky et al., 2006). The orally administered drugs are exposed to gut bacteria that can potentially metabolise these drugs through their metabolic enzymes and can modify their pharmacokinetic and pharmacodynamic properties (Haiser et al., 2014). This may result in variations in dietary and drug responses that are distinctive to individuals or populations due to variations in gut microbial communities between different individuals and populations(Sharma et al., 2017). Such promiscuous metabolisms can lead to drug inactivation, generation of toxic by-products as well as conversion of prodrug into its active metabolite (Carmody and Turnbaugh, 2014; Lindell et al., 2022). Hence, it is imperative to understand the species-specific metabolism of biotic and xenobiotic molecules to explore the possible advantageous or detrimental impacts on the human host.

There have been numerous reports of gut bacteria-mediated biotransformation of drugs such as digoxin (Haiser et al., 2013; Kumar et al., 2018), acetaminophen (Clayton et al., 2009), amphetamine (Kumar et al., 2019), gemcitabine (Geller et al., 2017), *etc.*, resulting in variations in drug responses amongst different demographics. Similarly, the metabolism of undigested dietary components and bioactive molecules by gut bacteria can lead to the formation of bacterial secondary metabolites that can have beneficial effects on human health (Rossi et al., 2005). These distinct gut bacterial species can be directly targeted for therapeutic purposes and used as possible biomarkers for diagnosis and prognosis. Nevertheless, doing a thorough experimental analysis of each individual molecule by the gut microbiota using experimental methods is an arduous task due to the substantial longitudinal fluctuation and extensive phylogenetic variety of gut microbiota. Thus, a comprehensive database delineating the complex metabolism of pharmacological compounds would be highly beneficial for the community.

Currently, “Pharmacomicrobiomics” (Rizkallah et al., 2012), “Microbiota-Active Substance Interactions (MASI)” (Zeng et al., 2021), and “MagMD” (Zhou et al., 2022) are such databases available that contain information about how gut microbes break down drugs. Most of these databases provide only species-level biotransformation of molecules. In contrast, we aim to provide strain-level metabolism of molecules since the strain-level analysis offers a higher degree of precision and accuracy when examining microbial diversity and microevolution within a species. This approach also allows for a more comprehensive understanding of microbial communities than species-level analysis.

We have developed a comprehensive database entitled “GutBugDB” containing information about human gut bacteria-mediated metabolism of 1378 FDA-approved drugs as well as 61 known nutraceuticals and bioactive dietary components. This database provides researchers with a comprehensive resource of gut bacteria-mediated metabolite metabolisms that may have varying effects on drug efficacy, metabolism, or adverse reactions among individuals. GutBugDB is available at https://metabiosys.iiserb.ac.in/gutbugdb/.

## Materials and methods

### Collection and classification of all the FDA-approved drugs

Using the database for FDA-approved drugs (https://www.fda.gov/) and literature review, a list of 1439 drugs and nutraceuticals was compiled. DrugBank database (Wishart et al., 2018) was used to retrieve information regarding the physiological target and therapeutic applications of the selected drugs. Based on this information, the selected drugs were classified into 14 categories: drugs acting on autonomous nervous system, respiratory system drugs, drugs acting on peripheral nervous system, Cardiovascular drugs, drugs acting on blood and blood formation, Antimicrobial drugs, Autocoids and related drugs, chemotherapy of neoplastic diseases, drugs acting on central nervous system, drugs acting on kidney, gastrointestinal drugs, hormones and related drugs, nutraceuticals, and miscellaneous drugs (Table 1).

**Table 1.** Number of drugs and nutraceuticals classified and analysed in different categories

Pharmacological categories	Number
Drugs acting on autonomous nervous system	121
Autocoids and related drugs	119
Respiratory system drugs	36
Hormones and related drugs	141
Drugs acting on peripheral nervous system	36
Drugs acting on central nervous system	250
Cardiovascular drugs	116
Drugs acting on kidney	32
Drugs acting on blood and blood formation	48
Gastrointestinal drugs	33
Antimicrobial drugs	258
Chemotherapy of neoplastic diseases	81
Miscellaneous drugs	107
Nutraceuticals	61
Total	1439

### Prediction of gut bacteria-mediated biotransformation of selected drugs using GutBug

GutBug is a web-based tool that combines artificial intelligence, machine learning, and cheminformatics to predict all potential bacterial metabolic enzymes involved in the biotransformation of biotic and xenobiotic molecules (Malwe et al., 2023). It is trained on 3457 enzyme substrates to predict the EC number(s) of gut bacterial enzymes and gut bacterial strains harbouring them. GutBug tool has a modular design where the first module is used to predict the first digit of an EC number or reaction class, the second module is used to predict the second digit of an EC number or reaction subclass, and the third module is used to predict the complete EC number of enzymes. Module 1 utilises 12 mutually exclusive binary classification models developed using random forest and artificial neural networks, whereas, for Module 2, six multilabel random forest models are used for predicting reaction subclasses. Module 3 uses a molecular similarity search approach to obtain a complete EC number of enzymes that can potentially metabolize the molecule of interest. Using an integrated gut bacterial enzymes database containing EC number-tagged 363,872 enzymes from 690 gut bacterial strains, the predicted EC numbers are used to obtain gut bacterial strains harbouring the predicted enzymes. PubChem ID for all 1439 biotic and xenobiotic molecules included in GutBugDB was used as an input to obtain GutBug predictions.

## Construction of database

### Building web interface

**Figure 1.**
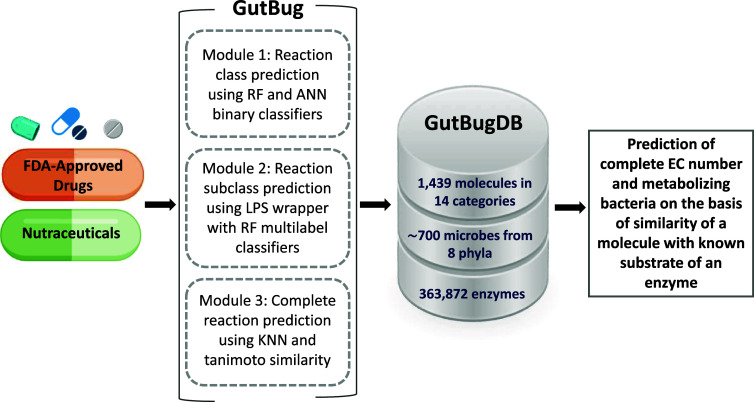
Overview of GutBugDB methodology.

A user-friendly web interface of GutBugDB was developed using MySQL, PHP, HTML, and JavaScript. The relational database underlying the web portal was designed and built using MySQL. The complete workflow of GutBugDB is represented in Figure 1.

### Browse options for GutBugDB

The web-based interface includes three basic search options.

#### Search by drug category

Users can perform a search by any of the previously mentioned pharmacological categories, which will display a list of drugs in the particular category. From this list, the users can select the drug of their interest, which will provide detailed information about the drug. The output page provides drug information on the selected drug that includes the drug name, pharmacological category, description, usage, DrugBank ID, PubChem ID, chemical formula, chemical structure, molecular weight, and IUPAC name of the drug. Further, an option is provided to select the Tanimoto threshold value to assess the similarity of the drug with a known bacterial metabolic substrate as calculated by GutBug. The default value is kept as 0.6 since at this or above value, a reliable prediction is expected; however, the user can select value below this threshold also if no hits are found at the default threshold for a submitted query. The “Predicted EC number and metabolising bacteria” section displays information on the predicted enzyme and bacteria capable of metabolizing the drug. The “Taxonomic information of predicted genus” section provides taxonomic information of the genus of the predicted bacterial species that can metabolize the drug including genus count and pie charts of family and genus distribution of bacterial species that can metabolize the selected drug molecule. Finally, under the “Prediction results of GutBug” section, the predicted metabolism information including enzyme and bacterium for the selected drug is provided.

#### Search by gut bacteria

Users can perform a search by the name of the phylum and it will display a list of all the gut bacterial strains that fall under that particular phylum. The user can click on the bacterial strain of interest to list out all the drug molecules that could be metabolized by the selected strain. Detailed metabolic information about any of the listed drugs can be retrieved by clicking on the same. A tutorial for navigating through GutBugDB is provided in the “Tutorial” section available on the web server (https://metabiosys.iiserb.ac.in/gutbugdb/tutorial.php).

#### Search by molecule name, PubChem ID, and bacterium name

Users can also search a drug name or PubChemID or bacterium name of interest using the search engine provided in the web server. The searched query will be displayed on the web page, where the user can click to get more information about the drug or bacteria. At the top of each table, an option is provided to download the results in csv format for the submitted query.

## Results and discussion

### Database overview

GutBugDB consists of 1439 molecules that include dietary bioactive components, nutraceuticals, and FDA-approved drugs classified into 14 categories based on their therapeutic applications. A Tanimoto similarity coefficient greater than or equal to 0.6 can be selected to get reliable predictions, resulting in a total of 214 molecules (Table 1). GutBugDB is highly enriched in bacterial metabolic and taxonomic information including 363,872 bacterial enzymes from 690 gut bacterial strains belonging to 8 phyla, 85 families, and 176 genera. These enzymes are tagged with their respective EC numbers and representative Expasy IDs and functional domains.

The information, features, and utility of the GutBugDB database were compared against the available Pharmacomicrobiomics database, MASI database, and MagMD database (Table 2). GutBugDB is constructed using machine learning-based predictions from GutBug, and by incorporating information from the previous studies. It has a comprehensive dataset of 1378 xenobiotic chemicals and 61 biotic molecules. The Pharmacomicrobiomics database contains drug–microbiome interactions for more than 60 drugs and was constructed using published literature. The MASI database was also constructed using published literature with a total of 1350 unique substances, including drugs, dietary, herbal, prebiotics, and environmental substances. Whereas the MagMD was constructed using previously available databases, published literature, and BLASTP with a total of 219 substances. Besides this, there is no information regarding FDA-approved drugs in the Pharmacomicrobiomics database, while the MASI database contains 980 approved drugs, and MagMD contains 123 FDA-approved drugs. On the other hand, GutBugDB contains 1378 FDA-approved drugs and 61 nutraceutical molecules and provides comprehensive information on the metabolism of these molecules as the result. Enzymes involved in xenobiotic metabolism are not reported in the Pharmacomicrobiomics and MASI database, and the MagMD database contains a total of 36 enzymes, whereas GutBugDB contains a total of 363,872 enzymes involved in the biotransformation of biotic and xenobiotic molecules.

**Table 2.** Performance of GutBugDB on validation set consisting of biotic and xenobiotic molecules

Molecule	Pharmacological category	Enzyme from literature	Enzymes reported by GutBugDB	Gut bacterial genera from literature	Gut bacterial genera reported by GutBugDB	EC number reported by GutBugDB
Biotic molecules						
Daidzen	Nutraceuticals	Reductase	Styrene monooxygenase, dichlorophenol monooxygenase	*Hugonella, Senegalimassilia*	*Achromobacter, Brevibacterium, Rhodococcus*	1.14.14.11, 1.14.13.20
Rutin	Nutraceuticals	α-l-Rhamnosidase	Thymidine phosphorylase, zeaxanthin glucosyltransferase	*Bifidobacterium*	*Bifidobacterium, Clostridium, Acinetobacter*	2.4.1.4, 2.4.1.276, 2.4.2.4
Lactulose	Gastrointestinal drug	Phosphorylase, Hydrolase	Glycosyl hydrolase, phosphorylases	*Bacteroides, Lactobacillus, Clostridium*	*Ruminococcus, Lactobacillus, Escherichia*	2.4.1.58, 2.4.2.10, 2.4.2.8
Inulin	Nutraceuticals	Fructanohydrolase	Fructofuranosidase, alpha-D-fructohydrolase	*Bifidobacterium, Klebsiella, Clostridium*	*Bifidobacterium, Lactobacillus, Clostridium, Ruminococcus*	3.2.1.22, 3.2.2.24, 3.2.1.74
Lacto-N-neo-tetraose	Nutraceuticals	N-acetylhexosaminidase, galactosidase	Galactosidase, glucuronidases, fructofuranosidase	*Bifidobacterium*	*Bifidobacterium, Roseburia, Enterococcus, Lactobacillus*	3.2.1.74, 3.2.1.67, 3.2.1.22
Lactosucrose	Nutraceuticals	NA	Sialidase, Glycoside hydrolase, beta-fructofuranoside	*Bifidobacterium, Blautia, Ruminococcus*	*Bifidobacterium, Ruminococcus, Blautia, Clostridium*	3.2.1.107, 3.2.1.122, 3.2.1.93
2- Fucosyllactose	Nutraceuticals	Glucosidases, fucosidase	6-Phospho-glucosidase, galacturonidase, phosphotrehalase, cellobiosidase	*Bifidobacterium*	*Bifidobacterium, Lactobacillus, Clostridium*	3.2.1.91, 3.2.1.107, 3.2.1.18
Xenobiotic molecules						
Lovastatin	Drugs affecting blood and blood formation	NA (hydroxylation)	Tyrosinase	NA	*Pseudomonas, Rhodococcus, Gordonia*	1.14.18.1
Sorivudine	Miscellaneous	NA (Hydrolysis)	Glycerol kinase	*Bacteroides*	*Parabacteroides, Clostridium, Enterococcus*	2.7.1.30
Hydrocortisone	Hormones and related drugs	Keto-reductase	Phthalate dioxygenase reductase, pyruvate hydroxylase	*Clostridium, Ruminococcus, Blautia, Dorea*	*Clostridium, Prevotella*	1.14.14.12, 1.14.99.48, 1.14.18.1, 1.14.15.15
Amphetamine	Central nervous system drug	Hydroxylase, monooxygenase, dioxygenases, Tyrosine oxidase	Benzoyl-CoA epoxidase, salicylyl-CoA hydroxylase, benzoate dioxygenase	*Lactobacillus, Clostridium, Enterococcus*	*Bradyrhizobium, Achromobacter, Streptomyces*	1.14.13.208, 1.14.13.209
Misoprostol	Autocoids and related drugs	NA (ester hydrolysis)	Phenylacetone monooxygenase, oxidoreductase	NA	*Rhodococcus, Bradyrhizobium*	1.14.13.92, 1.14.12.3, 1.14.14.52
Flucytosine	Chemotherapy of neoplastic diseases	NA (Deamination)	Cytosine deaminase	*Escherichia*	*Escherichia, Clostridium, Bifidobacterium*	3.5.4.1
Simvastatin	Drugs affecting blood and blood formation	NA (hydroxylation/β-oxidation)	Tyrosinase	*Lactobacillus*	*Streptomyces, Priestia*	1.14.18.1
Tacrolimus	Miscellaneous drugs	NA (Ketone reduction)	Uridine phosphorylase	*Faecalibacterium*	*Clostridium, Providencia, Klebsiella*	2.4.2.3, 2.4.1.346
Levodopa	Drugs acting on central nervous system	NA (Dehydroxylation)	Phenol 2-monooxygenase, 6-hydroxy–3-succinoylpyridine 3-monooxygenase	*Helicobacter*	*Acinetobacter, Delftia*	1.14.13.7, 1.14.13.163
Aspirin	Miscellaneous drugs	NA	Cholestanetriol 26-monooxygenase	NA	*Streptomyces, Bradyrhizobium*	1.14.15.15

### Validation dataset used for GutBugDB

**Figure 2.**
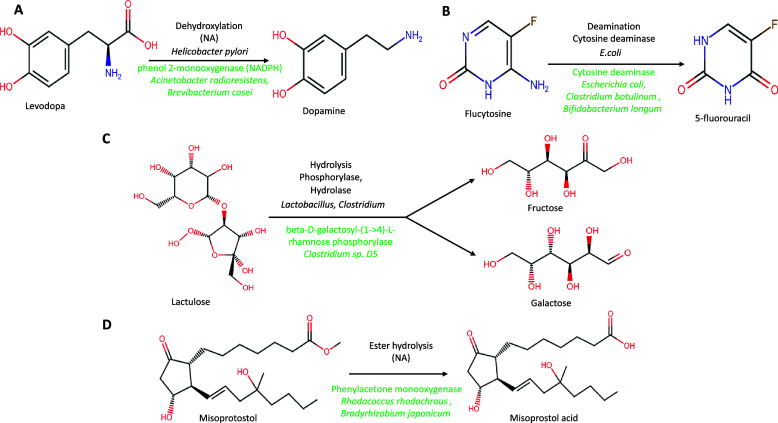
Human gut bacteria-mediated biotransformation of (A) levodopa, (B) flucytosine, (C) lactulose, and (D) misoprostol. The black colour font above the arrow represents biotransformation information as available in the literature and the green represents the predictions of GutBugDB.

The validation was performed on a set of seven biotic and 10 xenobiotic molecules that were experimentally shown to undergo human gut bacteria-mediated biotransformation. These molecules were used to validate the metabolic and biotransformation information provided by GutBugDB (Table 3, Figure 2). Similarly, in many experimentally identified metabolisms of orally ingested molecules, the type of reactions is understood but either the gut bacterium or enzymes causing the biotransformation are not yet known. Such examples were also included in the validation to highlight the utility and importance of the information compiled in GutBugDB.

**Table 3.** Comparison of GutBugDB with previously available databases

Features	Pharmacomicrobiomics	MASI	MagMD	GutBugDB
Number of molecules^a^	60+	1350	219	1439
FDA-approved	NA	980	123	1378
Pharmacological categories	No classification of drugs	Drugs classified into 5 categories	No classification of drugs	Drugs classified into 14 pharmacological categories
Enzymes	Enzymes involved in metabolism are not included	NA	36	363,872
Biotic and xenobiotic molecules	Only xenobiotic molecules	1074 xenobiotic and 72 biotic molecules	NA	1378 xenobiotic and 61 biotic molecules

a Includes drugs, nutraceuticals, substances*, etc.*

In the case of biotic molecules such as rutin, inulin, 2-fucosyllactose, and xenobiotic molecules such as hydrocortisone, amphetamine, *etc.*, GutBugDB provides information on gut bacteria and enzymes known in the literature, along with novel enzymes and gut bacterial strains previously not reported. For example in the case of Lactulose, a non-absorbable sugar that is used to treat hepatic encephalopathy and constipation, hydrolysis of lactulose into fructose and galactose, is catalysed by the phosphorylase and hydrolase enzymes of the gut bacteria *Lactobacillus*, *Bacteroides*, and *E. coli* (Sahota et al., 1982; Jourova et al., 2016). GutBugDB has the same enzymes and gut bacteria that can metabolise lactulose, with additional gut bacterial strains belonging to *Ruminococcus* and *Escherichia* that can also potentially metabolise lactulose (Table 3, Figure 2).

GutBugDB also contains biotransformation information for molecules like lactosucrose, sorivudine, and l-DOPA for which there is a lack of information about bacterial enzymes involved in their biotransformation. l-DOPA (l-3,4-dihydroxyphenylalanine), when administered orally, undergoes *Helicobacter-*mediated dehydroxylation of the catechol ring of l-dopa, forming the metabolites Dopamine and Serotonin (Goldin et al., 1973), with the enzyme involved in its dehydroxylation still unknown. GutBugDB predicted phenol 2-monooxygenase and 6-hydroxy-3-succinoylpyridine 3-monooxygenase enzymes from bacteria belonging to *Acinetobacter* and *Delftia* genera that can metabolise l-DOPA, thus identifying novel gut bacteria as well as enzymes involved in l-DOPA biotransformation. Similarly, the case of flucytosine, which is a fluorinated pyrimidine analogue and an antifungal drug made in a lab, is another interesting example where the bacterial enzymes turn flucytosine into 5-fluorouracil; however, the enzyme has not yet been identified. GutBugDB provides information that gut bacteria belonging to *Escherichia*, *Bifidobacterium*, and *Clostridium* genera have cytosine deaminase enzymes that can break down flucytosine.

In the case of misoprostol and aspirin, preliminary reports indicating human gut microbiome-mediated biotransformation are available, but neither the gut bacteria nor the enzymes involved in their metabolism have been identified (Zhang et al., 2019; Javdan et al., 2020). GutBugDB provides more information regarding misoprostol biotransformation by identifying monooxygenase and oxidoreductases present in *Rhodococcus* and *Bradyrhizobium* species that can potentially metabolise misoprostol. These results are in agreement with the studies indicating gut microbiome-mediated ester hydrolysis of misoprostol (Javdan et al., 2020). GutBugDB provided novel information on aspirin biotransformation by identifying cholestanetriol 26-monooxygenase present in *Bradyrhizobium*, thus indicating the potential hydrolysis of aspirin.

The performance of GutBugDB was also compared against the available Pharmacomicrobiomics database, MASI database, and MagMD database using the examples included in the validation set. Out of the 17 biotic and xenobiotic molecules in the validation dataset, eight molecules could be found in the Pharmacomicrobiomics database, 12 in the MASI database, and 12 were also present in the MagMD database for the comparative analysis (Table 3). Moreover, it was noted that Pharmacomicrobiomics and MagMD were more focused on drug molecules, whereas GutBugDB and MASI contained biotic as well as xenobiotic molecules, among which GutBugDB contained a comparatively much larger number of biotic and xenobiotic molecules along with the associated information. Lastly, Pharmacomicrobiomics and MagMD provide information about the known metabolising bacteria but lack information about metabolising enzymes, whereas GutBugDB provides information on the predicted enzymes and bacteria that can potentially metabolise a variety of biotic and xenobiotic molecules in addition to the known examples (literature) that were also accurately predicted.

## Conclusion

Recent studies have demonstrated the significance of the HGM in the metabolism of orally ingested biotic as well as xenobiotic molecules. The gut microbiome-mediated biotransformation of such molecules impacts the pharmacokinetics of drugs and nutraceuticals, affecting their bioavailability, efficacy, or toxicity. However, the role of gut microbiome-mediated biotransformation is often not determined during the drug development process due to the time-consuming and additional costs involved. Therefore, we developed GutBugDB to provide detailed information about the potential metabolism of drugs and nutraceuticals by human gut bacteria and their metabolic enzymes. The information provided in GutBugDB can be useful in identifying potential biotransformation of candidate drug molecules as well as during drug prescription to prevent drug non-responsiveness and to improve the effectiveness and tolerability of medications. The inclusion of information regarding the biotransformation of biotic molecules also helps in the formulation and prescription of nutraceuticals. The information contained in GutBugDB also provides leads for further experimental validations, which still remains the gold standard for conclusively identifying gut bacteria-mediated biotransformation of biotic and xenobiotic molecules. GutBugDB is scheduled for regular updates, with the last update on June 2024, and is compatible for the integration of new data on gut bacterial species and drug molecules from the forthcoming metagenomic studies.

## Data Availability

The data of this study is available online using GutBugDB database at https://metabiosys.iiserb.ac.in/gutbugdb.
